# A Bayesian Assessment of Real-World Behavior During Multitasking

**DOI:** 10.1007/s12559-017-9500-6

**Published:** 2017-08-12

**Authors:** Jeroen H.M. Bergmann, Joan Fei, David A Green, Amir Hussain, Newton Howard

**Affiliations:** 1 Institute of Biomedical Engineering, Department of Engineering Science, Oxford Natural Interactions Lab, Old Road Campus Research Building, University of Oxford, Oxford, UK; 2 Massachusetts Institute of Technology, Boston, USA; 3 Centre of Human & Aerospace Physiological Sciences, King’s College London, London, SE1 1UL UK; 4 KBRwyle, European Astronaut Centre, Linder Höhe, 51147 Cologne, Germany; 5 Division of Computing Science & Maths, School of Natural Sciences, University of Stirling, Stirling, FK9 4LA UK; 6 Nuffield Department of Surgical Sciences, University of Oxford, Oxford, UK

**Keywords:** Wearable sensors, Activities of daily living, Cognitive loading, Executive function, Motor control

## Abstract

Multitasking is common in everyday life, but its effect on activities of daily living is not well understood. Critical appraisal of performance for both healthy individuals and patients is required. Motor activities during meal preparation were monitored in healthy individuals with a wearable sensor network during single and multitask conditions. Motor performance was quantified by the median frequencies (*f*_m_) of hand trajectories and wrist accelerations. The probability that multitasking occurred based on the obtained motor information was estimated using a Naïve Bayes Model, with a specific focus on the single and triple loading conditions. The Bayesian probability estimator showed task distinction for the wrist accelerometer data at the high and low value ranges. The likelihood of encountering a certain motor performance during well-established everyday activities, such as preparing a simple meal, changed when additional (cognitive) tasks were performed. Within a healthy population, the probability of lower acceleration frequency patterns increases when people are asked to multitask. Cognitive decline due to aging or disease might yield even greater differences.

**Electronic supplementary material:**

The online version of this article (doi:10.1007/s12559-017-9500-6) contains supplementary material, which is available to authorized users.

## Background

Much can be learned about the brain by studying motor coordination [1]. Motor behavior, defined as the combination of movements that produce purposeful or intended actions, emerges due to a synergy between a range of systems [2]. The systems involved in this behavior are bounded by certain parameters and they have evolved to work within real-world constrains. Everyday living activities arise through the complex interaction of these factors and dysfunction within these factors will generate alternative behaviors. The occurrence of large changes in everyday living behavior can be an indicator that (patho)physiological changes are emerging. This is also the reason that at present, the diagnosis of disorders such as Alzheimer’s disease still heavily depend on the clinical history [3] and the observed behavioral changes by relatives and friends. Furthermore, there is growing evidence that indicates a link exists between activities of daily living (ADL) and executive dysfunction in patients suffering from early dementia [4].

It is unclear how changes in certain parameters might affect the behavior under real-world conditions. The complex interactions underlying behavior can be better understood by e.g., exploring effects of cognitive loading in a healthy populations. This information will be particularly interesting if it represents behavior that is common in the real-world. To our knowledge, no detailed assessments of multitasking within an ADL context exists, in which the tasks cover a range of complex everyday tasks. It still remains ambiguous to what extent everyday living is affected by cognitive ability. Current cognitive loading experiments often consist of experimental designs that do not capture the real-world performance of the biological system as they would occur on a daily basis. Issues with this standard experimental approach have been highlighted in a paper that investigated postural prioritization during multitasking [5]. It showed that well-established principles of “posture first” strategies, wherein individuals favors execution of motor components over execution of cognitive components, degraded when measurements were taken in more complex environments. This kind of decrement in motor performance can have devastating real-world impacts if it leads to for example a fall or injury. To what degree our complex motor performance is affected by cognitive loading needs to be explored further.

In the case that complex ADL motor performance is affected by cognitive loading then logic would imply that we might be able to predict cognitive loading by monitoring motor behavior itself. Studies have been performed on more skilled tasks such as handwriting [6], but activities that stretch over several phases which make up a complex everyday task have not been researched under multitasking conditions. In this explorative study, motor performance is investigated, while variable levels of cognitive loading are introduced. The probability that multitasking occurred based on motor performance data (hand trajectories and wrist accelerations) will be estimated with a Naïve Bayes Model. The model is a simple probabilistic method based on Bayes Theorem. In practice, Naïve Bayes models often compete rather well compared to more sophisticated models [7]. The simplicity, large community of users and ease of implementation makes the Naïve Bayes Model an ideal candidate for initial exploration of real-world multitasking.

The aim of this study is to investigate to what extent meal preparation is influenced by multitasking. It is hypothesized that multitasking will increase the probability of observing “slower” motion patterns, as defined by a decrease in the median frequency.

## Methods

### Subjects

In total 21 (8 male, 13 female), healthy participants were recruited, with a mean age of 23 (±3) years, an average height of 170 (±8) cm, and an average weight of 67 (±12) kg. All participants gave written and informed consent to volunteer for the study and the protocol was approved by King’s College London BDM Research Ethics Subcommittee.

### Equipment

**Fig. 1:**
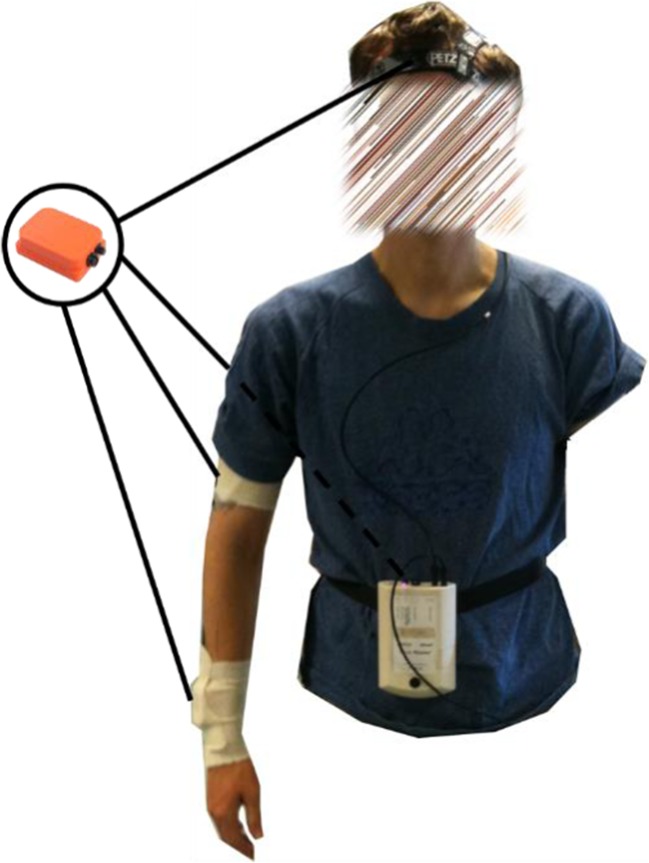
Experimental setup. Four inertial measurement units (sensors) were attached to the subject. They were placed just above the wrist, the upper arm, the lower back, and on the head

Participants wore a body sensor network (Xsens Technologies Ltd., The Netherlands). The network consisted of four sensors; they were attached to the right upper arm, right lower arm, head, and back (Fig. 1). The back sensor was used as reference sensor to determine if all other sensors worked appropriately.

Sensors on the arm and back were kept in place by double-sided tape and straps. The head sensor was placed on a non-slip elastic headband.

### Procedure

Subjects were asked to perform the task of preparing a meal during a 40-s trial. The meal preparation consisted of making as many sandwiches as possible and it was constructed to include several items from the Motor Activity Log (MAL) for the upper extremity [8]. Participants were asked to butter and cut as many slices of bread as possible within 40 s.

Subjects were instructed to speak freely and/or perform an additional cognitive activity (stroop task) during certain trials, while always performing the aforementioned everyday motor task. Four conditions were implemented and these consisted of just performing the motor task (single-task condition), performing the motor task while speaking (dual-task condition with speech) or while conducting a stroop task (dual-task condition with stroop task), and finally performing the motor activity concurrently with both speaking and a cognition task (triple task condition). The single task condition consisted of a trial at the start of the experiment and one at the end. A total of three trials were recorded for all other conditions. Conditions were pseudo-randomized in order to eliminate sequence effects in the outcomes.

### Stroop Task

The cognitive loading task consisted of a specific audio-spatial assignment. The auditory spatial task utilized a spatial stroop stimulus and it was presented through a wireless stereo headphone. Within one trial, three stimuli were given, with 10 s between each stimulus. The subjects were requested to response to unilateral aural stimuli. The stimuli consisted of the words “Left” and “Right” delivered through either the left or right headphone speaker. If the word matches the side, it was presented to (i.e., “Left” in the left ear) the result is congruous and therefore the appropriate response was to tell the researcher it was correct by shaking the head up and down. If incongruous, the subject is asked to state it was incorrect by shaking sideways.

Stimuli generation for the stroop task, as well as the data acquisition was performed in Matlab R2014a (MathWorks Inc., Natick, MA, USA).

### Stroop Task Response Detection

The head-mounted sensor was used to collect the angular velocities (°/s) in pitch direction (ω_pitch_; stating it was “correct”) and yaw direction (ω_yaw_; stating it was “incorrect”). The power spectral density *P*(*f*) was estimated for each direction (ω) using Welch’s method [9]. The method is based on applying a discrete Fourier transform (DFT, see Eq. 1) to estimate the power spectra, then splitting the data into windows, taking modified periodograms of these windows and finally averaging the obtained periodograms.

1 $$ {\varOmega}_{\mathit{\mathsf{k}}+\mathsf{1}}=\sum \limits_{\mathit{\mathsf{j}}=\mathsf{0}}^{\mathit{\mathsf{n}}-\mathsf{1}}{\left({\mathit{\mathsf{e}}}^{-\mathsf{2}\pi \mathit{\mathsf{i}}/\mathit{\mathsf{n}}}\right)}^{\mathit{\mathsf{j}\mathsf{k}}}{\omega}_{\mathit{\mathsf{j}}+\mathsf{1}} $$

The DFT equation (Eq. 1) takes in one of the head movement directions (ω_pitch_ or ω_yaw_) containing *n* sampled data points, with an index (*j*). Here, *i* is the imaginary unit and *k* the index to output *Ω.* A fast Fourier transform (FFT) was subsequently applied as a more efficient way of computing the required DFT. The frequency at which the power spectral density then reaches its maximum (*f*_maximum_) was compared against an expected relevant physiological range of .5–.10 Hz [10, 11]. Frequencies outside this range were assumed as unlikely voluntary physiological responses and labeled as “no response given.” All signals were checked for a potential second peak whenever the initially detected peak fell outside the physiological range. This approach was taken in order to prevent incorrect dismissal of data. The continuous wavelet transform was computed for all signals that showed *f*_maximum_ within the selected range. It was assumed that the nodding response would be best represented by a Morlet wavelet. This wavelet is the product of a complex exponential wave and a Gaussian envelope. The Morlet wavelet’s function *ψ*(*t*) is taken from [12] and can be described by

2 $$ \psi (t)={\mathrm{e}}^{\frac{-{\beta}^2{t}^2}{2}}\cos \left(\pi t\right) $$

in which *t* is time with *β* controlling the shape by balancing the time and frequency resolution. The following descriptions of the wavelet equations are adapted from [13, 14]. The Morlet wavelet can be defined as a “mother” wavelet from which a range of wavelets can be generated by scaling and translating,

3 $$ {\psi}_{a,b}(t)=\frac{1}{\sqrt{a}}\bullet \psi \left(\frac{t-b}{a}\right)\kern1.75em \mathrm{for}\ a>0, b\epsilon \mathit{\mathbb{R}} $$

in which *a* is the scaling parameter and *b* is the translation parameter, with *t* denoting the independent variable. The collection of wavelets that arise from this can be used as an orthonormal basis. The relevant coefficients can be obtained by

4 $$ {C}_{a,b,f(t),\psi }={\int}_{-\infty}^{\infty }f(t)\bullet {\psi}_{a,b}\left(\mathrm{t}\right) dt $$

Varying the values of *a* and *b* will provide the continuous wavelet transform coefficients *C*_*a,b*_ indicating how closely the wavelet is correlated to the original signal. These coefficients are of course dependent on the selected waveform (*ψ*) and function (*f*). A larger value for *C*_*a,b*_ shows a greater similarity between *ψ* and *f.*

**Fig. 2:**
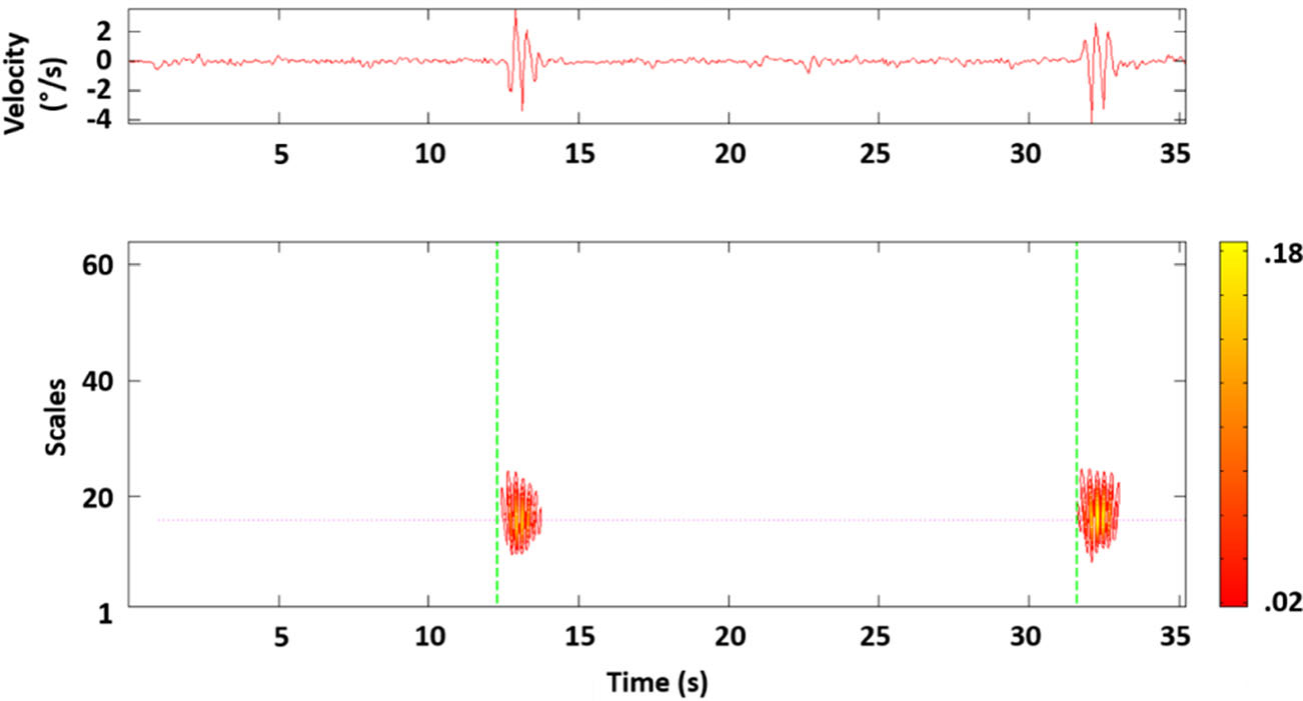
Example of stroop task response detection based on energy percentage of each wavelet coefficient. *Top* figure shows the original angular velocity signal in yaw direction across time. *Bottom* figure shows the scalogram of wavelet coefficients. It provides the percentage of energy for each coefficient depicted by a heat map that is given on the side. *Dotted green lines* show identified crossings of the set threshold (*dotted purple line*)

A scalogram of wavelet coefficients was then generated. The start of a specific response was defined as the point when the energy level of the *f*_max_ scale crossed a pre-set boundary. A limitation with applying a single value crossing is the selection bias. In order to overcome this as much as possible a range of thresholds were explored by

5 $$ {T}_{\mathrm{current}}=\frac{E_{\mathrm{max}}}{T}\kern1.50em \left\{T\in \mathbb{N}|1\le T\le 100\right\} $$

with *E*_max_ being the maximum energy and *T* the threshold denominator set to produce a current threshold (*T*_current_). Analysis of pilot data indicated that large shifts could be minimized when a *T* of 22 was applied. To allow for some random variation, *T* was set to 30. This gave the following formula to detect within a 10-s interval the first energy (*E*) crossing by

6 $$ E>\frac{E_{\mathrm{max}}}{30} $$

the equation showed good identification of responses across several pilot test sessions. An example is shown in Fig. 2.

The time at which a certain stimulus was given was subtracted from the time when a response was detected. This value represented the response time of the subject. A window size of 10 s was used to identify any responses, as the stimuli were generated at a .1 Hz rate. The response was labeled “incorrect” if no response was found. Identified responses were compared to the expected response. If the response was expected to occur within a specific direction (yaw or pitch) the response was labeled “correct.” Otherwise, the response was deemed “incorrect.”

However, it could be that there is a response signal present in both yaw and pitch direction. In this case, it needs to be determined if a corrective action (yaw and pitch response are separated in time) has taken place or if it is crosstalk of the channels due to for example rigorous shaking. Crosstalk is defined as one signal overlapping the other and can be formalized as:

7 $$ {t}_{\mathrm{yaw}(1)}<{t}_{\mathrm{pitch}(n)}\bigwedge {t}_{\mathrm{pitch}(1)}<{t}_{yaw(n)} $$

In which *t*_yaw (1)_ and *t*_pitch (1)_ are the time points at the start of the response and *t*_yaw(n)_ and *t*_pitch(*n*)_ are indicating the end of the response. If any overlap is detected, the signal with the highest average energy is identified as the leading signal (1 is assigned) and the other signal is seen as the crosstalk signal (assigning it a value of .5). If both signals are equal in terms of average energy, they are both assigned a value of .5 and it can be stated that it is inconclusive which response the subject wanted to give.

A truth matrix consisting of dichotomized outcomes allows for easy assessment of performance. The first two cells of each row can be summed and if this value is greater than 1 the performance can be labeled as correct. This simple computation provides a quick top level view of the provided responses. The summed outcomes were labeled as extracted responses. Response detection was further validated during a small pilot trial (see supplementary information).

### Motor Performance

Upper limb motion patterns were obtained through a simple biomechanical model [15]. The Euclidian norm of the hand trajectory was computed by

8 $$ \left\Vert p\right\Vert =\sqrt{p_x^2+{p}_y^2+{p}_z^2} $$

with *p* as the 3D position vector [*p*_*x*_ *p*_*y*_ *p*_*z*_]. This norm was computed for each index point and used for further analysis. This norm seems to differentiate well between everyday motions [15]. Another more “practical” method used the median frequency (*f*_m_) of the acceleration norm (‖*a*‖) [16], which was obtained from wrist sensor. The *f*_m_ was defined as,

9 $$ \frac{1}{2}{\int}_0^{f_{\mathrm{max}}}P(f) df $$

with *f* being the frequency in Hz, *f*_max_ the maximum frequency in the spectrum and *P*(*f*) the power spectral density. Median frequency was computed for both ‖*p*‖ and ‖*a*‖ for a 3-s block that was taken directly after the stroop task stimulus was applied. For the unloaded condition, a 3-s data block was taken at similar time intervals. All three *f*_m_ within a trial were used to compute an average value representing trial performance. A detailed data flow diagram for this study is provided in the supplementary information.

## Statistical Analysis

A total of four trials could not be analyzed due to data corruption (two single and two multitask trials). These trials were therefore excluded from further statistical testing.

**Fig. 3:**
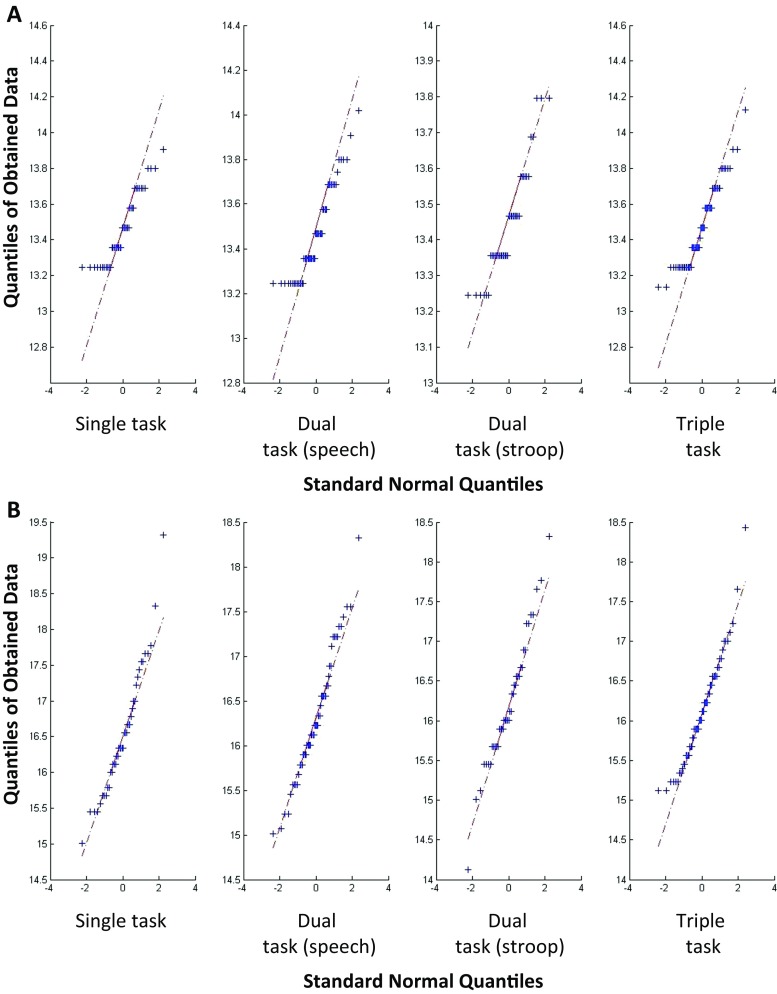
Q-Q plots showing the data across the four conditions for hand trajectories (**a**) and accelerations (**b**)

The Kolmogorov-Smirnov test [17] showed that median frequency (*f*_m_) data was not normally distributed (*p* < 0.01) for both the hand trajectories and accelerations. The test compares the empirical cumulative distribution function of the collected data with the expected normal distribution, with a significant result indicating that the data is not normally distributed. Q-Q (quantile-quantile) plots further confirmed a non-Gaussian distribution with zero mean and unit variance [18]. The Q-Q plots are used to visually check for normality (Fig. 3).

Boxplots were used to visualize the data. A rank transformation procedure was used in order to apply an analysis of variance on the data [19], with groups consisted of the four conditions (single task, dual with speech task, dual with stroop task, and triple task). The ranked *f*_m_ was used as the dependent variable. Subsequently, a non-parametric Kruskal-Wallis tests were performed upon acceleration and position data to establish if any differences were present between conditions [20].

It is likely that performance outcome (*f*_m_) follows a less ordered function. In order to explore this, a Naïve Bayes approach was applied on the task limits, i.e., single and triple task [21]. The Bayesian probability estimator used the predictors of hand trajectory *f*_m_ and acceleration *f*_m_ to classify between the single and triple task condition. A Kernel smoothing density estimator was applied for each predictor, as it was previously indicated that the data did not follow normality (see Fig. 3) and thus the density was estimated based on all the available data points. The prior probabilities are estimated from the relative frequencies of the single and triple task condition. The input feature matrix (*x*) consists of *f*_m_ columns for the hand position and acceleration, with *C*_*i*_ representing the two possible classes (*i = 1* for single task; *i = 2* for triple task), as described by Bayes’ Rule (Eq. 13).

10 $$ \mathit{\mathsf{P}}\left({\mathit{\mathsf{c}}}_{\mathit{\mathsf{l}}}|\mathit{\mathsf{x}}\right)=\frac{\mathit{\mathsf{P}}\left(\mathit{\mathsf{x}}|{\mathit{\mathsf{c}}}_{\mathit{\mathsf{l}}}\right)\mathit{\mathsf{P}}\left({\mathit{\mathsf{c}}}_{\mathit{\mathsf{l}}}\right)}{\mathit{\mathsf{P}}\left(\mathit{\mathsf{x}}\right)} $$

The probability that an observation belongs to a certain class (posterior probabilities) were estimated using the predictor space, which was defined by instances on a 2D–grid. The posterior probability that a classification is *C*_*i*_ for a given observation was computed by multiplying the conditional joint density of the predictors for a certain class with the class prior probability distribution and dividing it all by the joint density of the predictors [22]. It was hypothesized that a condition with an increased probability for lower *f*_m_ should yield a lower functional performance.

All data analysis and statistics were performed in Matlab R2014a (MathWorks Inc., Natick, MA, USA).

## Results

**Fig. 4:**
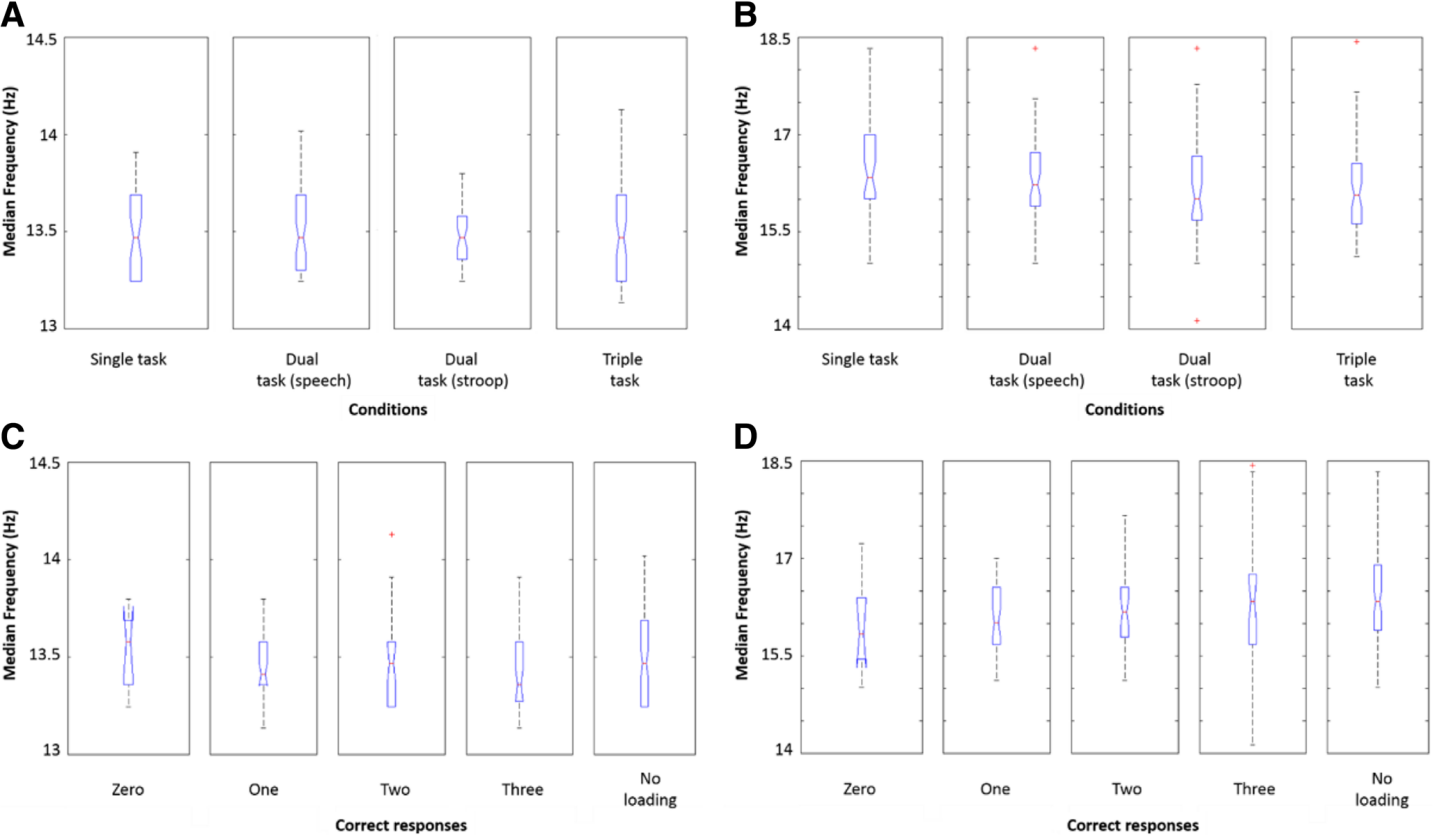
Boxplots of the median frequency across the four conditions for hand trajectories (**a**) and accelerations (**b**). Boxplots of the median frequency for trajectories (**c**) and accelerations (**d**) labeled by the total number of correct responses given for each trial. Trials that did not contain any stroop task was labeled as “no loading.” The median value is shown as the *central red mark* and the *edges of the box* representing the 25th and 75th percentiles. The *whiskers* represent the most extreme data points and *red crosses* are used for outliners

Boxplots were used to visualize the hand trajectories and accelerations between conditions (Fig. 4).

The ranked analysis of variance showed no significant difference between conditions for hand trajectory (*F*(3195) = 0.3170, *p* = 0.81) and acceleration (*F*(3195) = 2.556, *p* = 0.06). The Kruskal-Wallis test also found no significant differences for hand trajectory (*H*(3) = 0.246, *p* = 0.97) nor acceleration (*H*(3) = 6.852, *p* = 0.08).

**Fig. 5:**
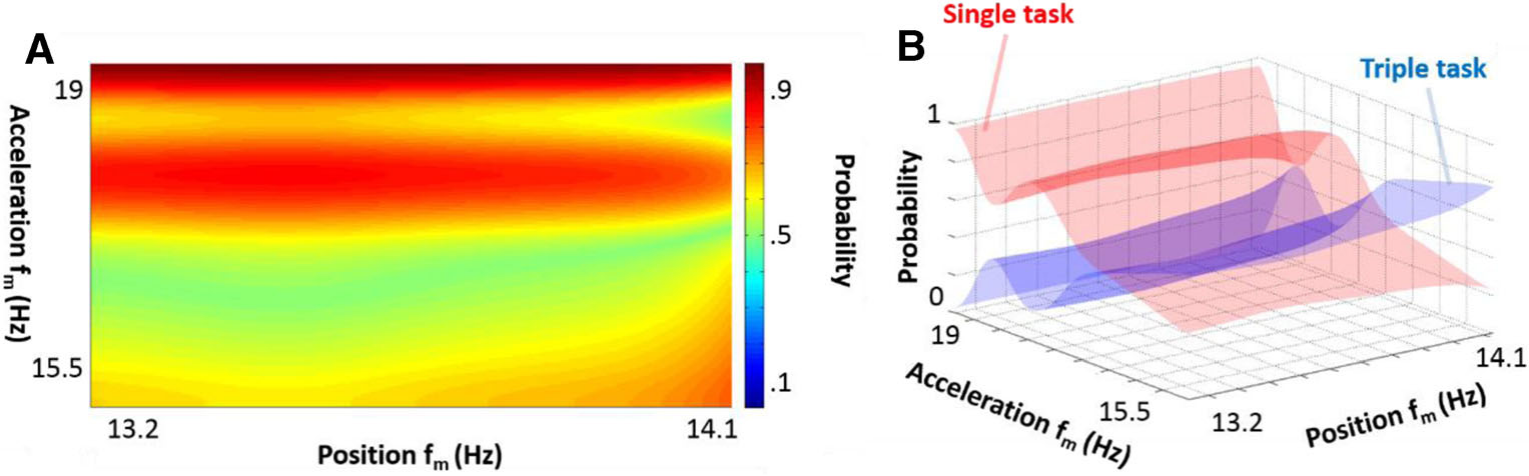
Visualizations of the estimated probability distribution between single and triple tasks. **a** Probability distribution between single and triple tasks, shown as heat map, given the features of *f*_m_ for position and acceleration. **b** Same probability distribution between single and triple tasks as shown in **a**, but now plotted in 3D for visualization purposes

The Bayesian probability estimator showed no clear task distinction based on the hand trajectory data. However, tasks could be differentiated based on the acceleration *f*_m_ values between the single and triple tasks (Fig. 5). The data indicates a clear distinction in the obtained *f*_m_ between single and triple task performance.

Applying this model for (same dataset) prediction would generate a misclassification of 38%, with most of the misclassification occurring in the *f*_m_ of acceleration region between 15.8 and 17.2 Hz covering the .4 to .6 range of task probability. This region contained 33% of all data points. Values outside this region yielded a relative good probability for separating the two tasks across all subjects. In general, higher *f*_m_ values for accelerations were found in the single task, while low values more likely indicated subjects performing a triple task.

## Discussion

Results showed no difference in hand trajectories between the conditions when traditional statistical methods such as the analysis of variance and Kruskal-Wallis test were used. However, visualization, analysis of variance and the Kruskal-Wallis test indicated a clear trend towards lower *f*_m_ for multitasking when the acceleration data was explored. The Bayesian probability estimator showed that differences existed in the probability estimates between the extremes (single and triple tasking). This differentiation between the single and triple task was also observed when the number of prepared sandwiches were counted. Participants completed less sandwiches when they were multitasking. It suggests that subjects will become “slower” both in *f*_m_ accelerations, as well as in overall functional performance, when they are requested to multitask. The motor differences appears to be too small to be subjectively perceived as a decline in performance by subjects, but they become apparent by applying a simple Naïve Bayes model. The *f*_m_ feature has previously been used to successfully classify different activities of daily living [16, 23], provides a relatively simple and thus informative metric. However, other features should be explored in order to determine if discrimination can be further improved.

Our human perception bias often exists in quantifying our own performance and this bias is also found in caretakers assessing activities of daily living in those who suffer from a decline in cognitive abilities [24]. A more objective approach to unobtrusively track function will therefore benefit both patients and clinical professionals. This kind of technology can especially impact those older adults who are living alone and the change attitude towards technologies can positively influence the uptake of these devices [25]. It is important to consider that the activities completed in this study are very natural and intuitive. Thus, the finding of any differentiation between single and multitasking in healthy activities of daily living is therefore a very intriguing and warrants further investigation.

Accelerations obtained from mobile devices have already been used for automatically detecting different states of physical activity [26]. Measuring wrist accelerations might therefore provide a smart and acceptable method for monitoring, as there is no need to define any task specific constrains. This data can be easily gathered by an unobtrusive wrist worn accelerometer. However, confirmation of the presented results in other datasets is still required.

Naïve Bayes models are very attractive for estimating general probability within real-time domains making it a suitable model for real-world tracking [21]. It also provides a computational inexpensive method for differentiating between tasks and it is relatively easy to implement. A key aspect of the model is that independence is assumed between the predictors, which may on occasions be violated. More sophisticated models should be applied in future studies to minimize assumptions, but these initial results show that even a simple method might be able to detect an increased probability of certain motor behavior occurring when healthy individuals start to multitask.

Although, real-world interaction is noisier, more heterogeneous and less repeatable than the induced stroop task, the induced task does reflect the domain of interest a lot better. The results found in this study seem comparable with other real-world scenarios. It has already been proven that mobile phone use has a detrimental influence on driving performance [27] and interestingly enough, even practice seems to be unable to eliminate the disruptive effects of concurrent cell phone use on driving [28]. Real-world multitasking, might therefore be strongly engrained into our behavior. This makes it easier to robustly monitor and assess any potential changes. It would also indicate that we should investigate cognitive load effects in the case of human-machine interfaces in order to make them more ecological valid.

This study shows that even simple everyday tasks performed by healthy individuals can be affected by multitasking for certain individuals. The potential to monitor this with an unobtrusive wearable sensor provides an interesting approach for further exploration in relevant patient populations, such as Parkinson’s disease (PD).

Parkinson’s disease (PD) is a progressive neurodegenerative disorder that affects the central nervous system and is primarily found in patients over 50 years of age. Symptoms include difficulty with motor skills such as walking and writing, as well as uncontrollable shaking (tremor), and general lethargy. These symptoms are caused by the death of neurons in the midbrain that control movement by generating dopamine, a neurotransmitter that modulates neural pathways and allows for smooth, controlled movement [29]. In later stages of the disease, patients may experience trouble with emotional control and dementia [29, 30]. Studies have shown that early movement impairments and cognitive deficits can provide insight into the underlying neurodegenerative processes [31]. In the case of Parkinson’s disease, changes in physical movement typically precede changes in language and behavior. Measurements of movement are therefore particularly valuable as indicators of the earliest stages of neural dysfunction. In addition, impairments in PD are exacerbated under simple dual-task conditions requiring the simultaneous performance of cognitive or motor tasks when compared to healthy controls [32–34]. This provides further evidence that the aforementioned method of monitoring ADL under a range of conditions might be able to accurately predict changes at the executive level.

## Conclusion

An increased probability of finding low median frequencies (*f*_m_) for wrist accelerations was found during complex multitasking compared a single activity. It shows that even in healthy individuals who are performing everyday tasks, changes can arise in motor performance due to multitasking. Differentiation based on probability is possible at the extreme ends of the recorded values, while overlap exists within the midrange. It is likely that certain patient populations will show even more pronounced differences in motor performance during multitasking. The opportunity to measure this with a modest wearable sensor makes it of interest for further research in relevant patient populations.

## Electronic Supplementary Material

ESM 1 (DOC 239 kb)
