# Supplementary material for: A Bayesian Assessment of Real-World Behavior During Multitasking
Source: Cognit Comput. 2017 Aug 12;9(6):749–57. doi: 10.1007/s12559-017-9500-6 (PMC5722954; doi:10.1007/s12559-017-9500-6)

**Validation of Response detection using wavelets**

Six experimental tests sets were generated, during which three were correctly answered and three incorrectly. Each test set consisted of three stimuli and three predetermined responses were given in return. The extracted responses (r_e_) from the outcome matrix were compared to the expected outcomes (r_exp_). The results show that across the test datasets no incorrect classification was made regarding the responses that were detected and those that were expected (Table 1).

***Table 1.*** *Validation results of classification algorithm between extracted (r_e_) and expected (r_exp_) outcomes. For the yaw and pitch signals a correct response is labelled 1, an incorrect response is labelled 0 and cross talk has a .5 value. The variables t_yaw(1)_ and* *t_pitch(1)_* represent the reaction time for a given response. Those that represent the reaction time of a correct response are given in bold. Dataset 1-3 contains only correct responses and 4-6 only incorrect.

| **Test**  **dataset** | **Stimulus** | **Yaw** | **Pitch** | **Sum yaw and pitch** | **r_e_** | **r_exp_** | **t_yaw(1)_(s)** | **t_pitch(1)_(s)** |
| --- | --- | --- | --- | --- | --- | --- | --- | --- |
| **1** | ***1*** | 1 | 0.5 | 1.5 | 1 | 1 | **4.18** | 3.26 |
|  | ***2*** | 1 | 0.5 | 1.5 | 1 | 1 | **3.02** | 2.96 |
|  | ***3*** | 1 | 1 | 2 | 1 | 1 | 0 | **2.74** |
| **2** | ***1*** | 1 | 1 | 2 | 1 | 1 | 0 | **3.24** |
|  | ***2*** | 1 | 0.5 | 1.5 | 1 | 1 | **2.76** | 2.7 |
|  | ***3*** | 1 | 0.5 | 1.5 | 1 | 1 | **3.8** | 3.8 |
| **3** | ***1*** | 1 | 0.5 | 1.5 | 1 | 1 | **3.12** | 3.12 |
|  | ***2*** | 1 | 1 | 2 | 1 | 1 | 0 | **2.68** |
|  | ***3*** | 1 | 0.5 | 1.5 | 1 | 1 | **3.08** | 4.84 |
| **4** | ***1*** | 0 | 0 | 0 | 0 | 0 |  |  |
|  | ***2*** | 0 | 0 | 0 | 0 | 0 |  |  |
|  | ***3*** | 0 | 0 | 0 | 0 | 0 |  |  |
| **5** | ***1*** | 0 | 0 | 0 | 0 | 0 |  |  |
|  | ***2*** | 0 | 0 | 0 | 0 | 0 |  |  |
|  | ***3*** | 0 | 0 | 0 | 0 | 0 |  |  |
| **6** | ***1*** | 0 | 0 | 0 | 0 | 0 |  |  |
|  | ***2*** | 0 | 0 | 0 | 0 | 0 |  |  |
|  | ***3*** | 0 | 0 | 0 | 0 | 0 |  |  |

**Data flow diagram**

The data flow diagram for generating output is provided below.


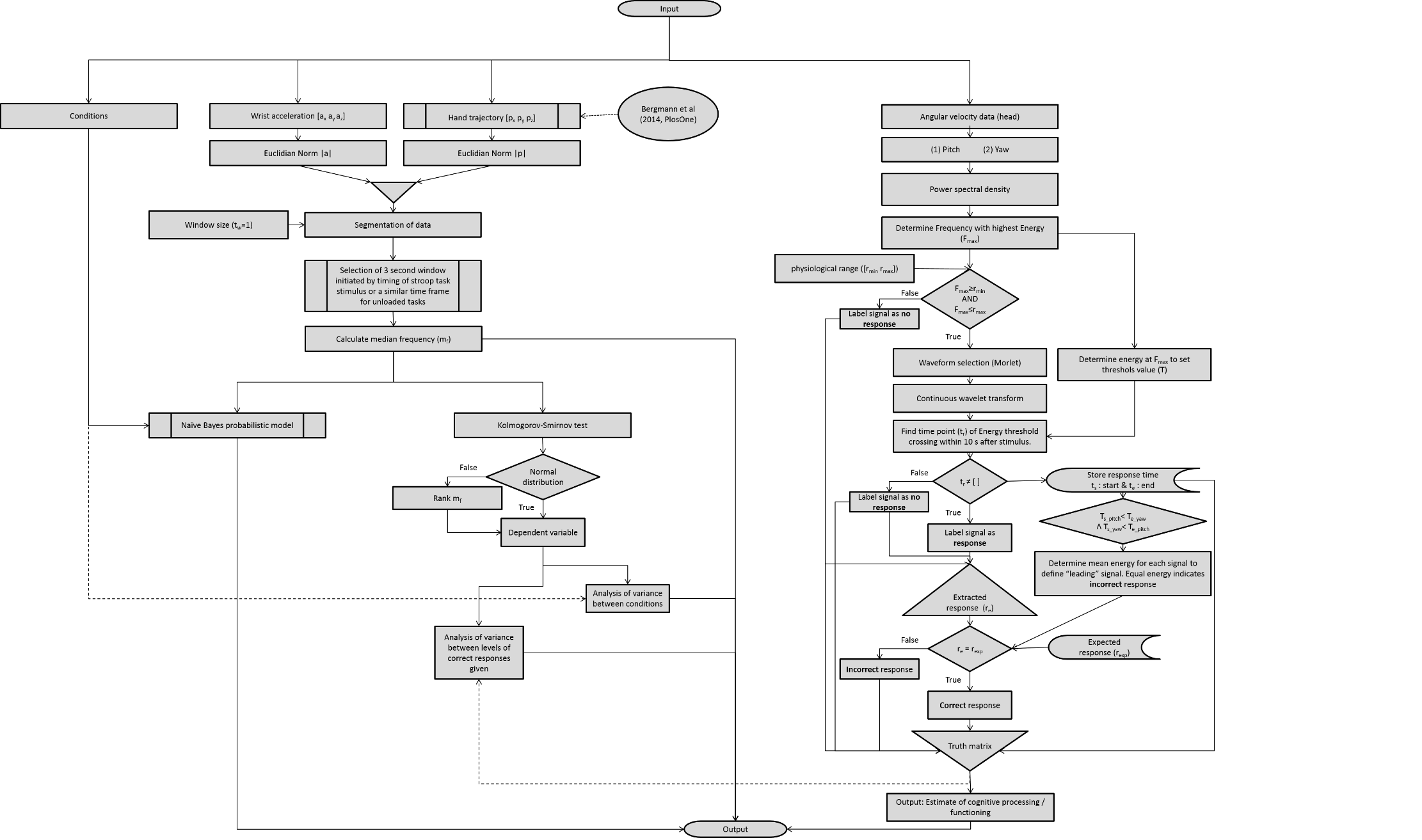

Supplement: Supplementary file 1 — (DOC 239 kb) [file 12559_2017_9500_MOESM1_ESM.doc]
